# Supplementary material for: Psychometric validation of an Arabic version of the WHO-5 wellbeing index among Lebanese adolescents
Source: PLoS One. 2025 Jul 17;20(7):e0317644. doi: 10.1371/journal.pone.0317644 (PMC12270098; doi:10.1371/journal.pone.0317644)
Supplement: S1 Table — Items for WHO-5, PHQ-9 and GAD-7. (DOCX) [file pone.0317644.s001.docx]

World Health Organization Wellbeing Index (WHO-5)

| الرجاء قراءة كل سؤال على حدة واختيار الإجابة الواحدة المناسبة التي تصف وضعك الخاص **خلال** الأسبوعين الماضيين**.** | | | | | | |
| --- | --- | --- | --- | --- | --- | --- |
| **كل الوقت /دائما** | **معظم الوقت** | **كثيراً من الوقت** | **بعض الوقت/ أحيانا** | **قليلاً من الوقت** | **أبدا** |  |
| 5 | 4 | 3 | 2 | 1 | 0 | 1) كم من الوقت كنت شخصاً سعيداً؟ |
| 5 | 4 | 3 | 2 | 1 | 0 | 2) كم من الوقت كنت تشعر بالهدوء والسلام؟ |
| 5 | 4 | 3 | 2 | 1 | 0 | 3) كم من الوقت كنت شخصاً مضطرباً؟ |
| 5 | 4 | 3 | 2 | 1 | 0 | 4) كم من الوقت كنت تشعر بكآبة وحزن في القلب؟ |
| 5 | 4 | 3 | 2 | 1 | 0 | 5) كم من الوقت كنت تشعر بالتعاسة لدرجة أن لا شيء يفرحك؟ |

Patient Health Questionnaire-9 (PHQ-9)

| خلال الاسبوعين الماضيين، ما مدى تكرار انزعاجك اثر اي من المشاكل التالية؟ | ابدا | عدة ايام | اكثر من  نصف الايام | تقريبا كل  يوم |
| --- | --- | --- | --- | --- |
| 1. فقدان المتعة والفرح في تأدية كافة الامور | 0 | 1 | 2 | 3 |
| 1. الشعور بالحزن، او الإكتئاب، أو اليأس | 0 | 1 | 2 | 3 |
| 1. اضطرابات في النوم (عدم القدرة على النوم، نوم متقطع او نوم زائد) | 0 | 1 | 2 | 3 |
| 1. الشعور بالتعب او بقلة الطاقة | 0 | 1 | 2 | 3 |
| 1. ضعف في الشهية او الافراط في تناول الطعام | 0 | 1 | 2 | 3 |
| 1. الشعور بالسوء حيال نفسك - و انك فاشل او انك قمت بخذل نفسك او اسرتك | 0 | 1 | 2 | 3 |
| 1. صعوبة في التركيز على الامور، مثل قراءة الجريدة او مشاهدة التلفاز | 0 | 1 | 2 | 3 |
| 1. التحرك او التكلم بغاية البطء بحيث قد يلاحظ على ذلك الاخرون. او عكس ذلك، ان تكون بغاية التململ او التهيج بحيث انك تتحرك اكثر بكثير من العادة. | 0 | 1 | 2 | 3 |
| 1. افكار حول انك ستكون افضل حالا لو كنت ميتا او ان تؤذي نفسك بطريقة ما | 0 | 1 | 2 | 3 |

Generalized Anxiety Disorder – 7 (GAD-7)

خلال الأسبوعين الماضيين، ما مدى تكرار إنزعاجك إثر أي من المشاكل التّالية؟ (ضع دائرة حول الرقم داخل المربّعات)

|  | | أبداً | عدّة أيّام | أكثر من نصف الأيّام | تقريباً كلّ يوم |
| --- | --- | --- | --- | --- | --- |
| 1 | الشعور بالتوتر، القلق، أو عدم الراحة | 1 | 2 | 3 | 4 |
| 2 | عدم القدرة على الامتناع أو السيطرة على القلق | 1 | 2 | 3 | 4 |
| 3 | القلق كثيراًً حول أمور عدّة | 1 | 2 | 3 | 4 |
| 4 | صعوبة في الاسترخاء | 1 | 2 | 3 | 4 |
| 5 | الشعور بالتململ لدرجة أنه من الصعب الجلوس في مكانك | 1 | 2 | 3 | 4 |
| 6 | من السهل اثارة غضبك أو انزعاجك | 1 | 2 | 3 | 4 |
| 7 | الشعور بالخوف وكأن شيئاً مريعاًً قد يحصل | 1 | 2 | 3 | 4 |
