# Supplementary material for: Psychometric validation of an Arabic version of the WHO-5 wellbeing index among Lebanese adolescents
Source: PLoS One. 2025 Jul 17;20(7):e0317644. doi: 10.1371/journal.pone.0317644 (PMC12270098; doi:10.1371/journal.pone.0317644)
Supplement: S2 Table — Items for WHO-5, PHQ-9 and GAD-7. (DOCX) [file pone.0317644.s002.docx]

World Health Organization Wellbeing Index (WHO-5)

| Please respond to each item by marking one box per row, regarding how you felt in the last two weeks. | | At no time | Some of the time | Less than half the time | More than half the time | Most of the time | All of the time |
| --- | --- | --- | --- | --- | --- | --- | --- |
| 1 | I have felt cheerful in good spirits. | 0 | 1 | 2 | 3 | 4 | 5 |
| 2 | I have felt calm and relaxed. | 0 | 1 | 2 | 3 | 4 | 5 |
| 3 | I have felt active and vigorous. | 0 | 1 | 2 | 3 | 4 | 5 |
| 4 | I woke up feeling fresh and rested. | 0 | 1 | 2 | 3 | 4 | 5 |
| 5 | My daily life has been filled with things that interest me. | 0 | 1 | 2 | 3 | 4 | 5 |

Patient Health Questionnaire-9 (PHQ-9)

| Over the last 2 weeks, how often have you been bothered by any of the following problems? | Not at all | Several days | More than half the days | Nearly every day |
| --- | --- | --- | --- | --- |
| 1. Little interest or pleasure in doing things | 0 | 1 | 2 | 3 |
| 2. Feeling down, depressed, or hopeless | 0 | 1 | 2 | 3 |
| 3. Trouble falling or staying asleep, or sleeping too much | 0 | 1 | 2 | 3 |
| 4. Feeling tired or having little energy | 0 | 1 | 2 | 3 |
| 5. Poor appetite or overeating | 0 | 1 | 2 | 3 |
| 6. Feeling bad about yourself — or that you are a failure or have let yourself or your family down | 0 | 1 | 2 | 3 |
| 7. Trouble concentrating on things, such as reading the newspaper or watching television | 0 | 1 | 2 | 3 |
| 8. Moving or speaking so slowly that other people could have noticed? Or the opposite — being so fidgety or restless that you have been moving around a lot more than usual | 0 | 1 | 2 | 3 |
| 9. Thoughts that you would be better off dead or of hurting yourself in some way | 0 | 1 | 2 | 3 |

Generalized Anxiety Disorder – 7 (GAD-7)

| Over the last two weeks, how often have you been bothered by the following problems? | Not at all | Several days | More than have the days | Nearly every day |
| --- | --- | --- | --- | --- |
| 1. Feeling nervous, anxious, or on edge | 1 | 2 | 3 | 4 |
| 1. Not being able to stop or control worrying | 1 | 2 | 3 | 4 |
| 1. Worrying too much about different things | 1 | 2 | 3 | 4 |
| 1. Trouble relaxing | 1 | 2 | 3 | 4 |
| 1. Being so restless that it is hard to sit still | 1 | 2 | 3 | 4 |
| 1. Becoming easily annoyed or irritable | 1 | 2 | 3 | 4 |
| 1. Feeling afraid, as if something awful might happen | 1 | 2 | 3 | 4 |
