# Supplementary material for: Psychometric validation of an Arabic version of the WHO-5 wellbeing index among Lebanese adolescents
Source: PLoS One. 2025 Jul 17;20(7):e0317644. doi: 10.1371/journal.pone.0317644 (PMC12270098; doi:10.1371/journal.pone.0317644)
Supplement: Attachment — Submitted filename: Response to Reviewers_WHO5_180325.docx [file pone.0317644.s003.docx]

**Dearest Editorial team**

**On behalf of my colleagues, we are grateful for the scientific rigorous comments provided to help uplift our manuscript and meet journal criteria**

**We are hereby providing a point-by-point reply to editor and reviewers in bold, and have highlighted in yellow tracked changes in the manuscript for ease of tracking**

**We look forward for the overall evaluation and are happy to be given the opportunity to give visibility to our work from the underrepresented part of the world.**

**Editor:**

1. Please ensure that your manuscript meets PLOS ONE's style requirements, including those for file naming.

**Done**

2. We note that the grant information you provided in the ‘Funding Information’ and ‘Financial Disclosure’ sections do not match.

When you resubmit, please ensure that you provide the correct grant numbers for the awards you received for your study in the ‘Funding Information’ section.

**Done**

3. Thank you for stating the following financial disclosure:

“This research was funded by Grand Challenges Canada – Global Mental Health – NIHS; R-GMH-POC-2107-47498.”

Please state what role the funders took in the study.  If the funders had no role, please state: "The funders had no role in study design, data collection and analysis, decision to publish, or preparation of the manuscript." If this statement is not correct you must amend it as needed.

Please include this amended Role of Funder statement in your cover letter; we will change the online submission form on your behalf.

**Done**

4. Please note that funding information should not appear in the Acknowledgments section or other areas of your manuscript. We will only publish funding information present in the Funding Statement section of the online submission form. Please remove any funding-related text from the manuscript.

**Noted. This has been processed accordingly.**

5. We note that you have indicated that there are restrictions to data sharing for this study. For studies involving human research participant data or other sensitive data, we encourage authors to share de-identified or anonymized data. However, when data cannot be publicly shared for ethical reasons, we allow authors to make their data sets available upon request. For information on unacceptable data access restrictions, please see http://journals.plos.org/plosone/s/data-availability#loc-unacceptable-data-access-restrictions.

Before we proceed with your manuscript, please address the following prompts:

a) If there are ethical or legal restrictions on sharing a de-identified data set, please explain them in detail (e.g., data contain potentially identifying or sensitive patient information, data are owned by a third-party organization, etc.) and who has imposed them (e.g., a Research Ethics Committee or Institutional Review Board, etc.). Please also provide contact information for a data access committee, ethics committee, or other institutional body to which data requests may be sent

b) If there are no restrictions, please upload the minimal anonymized data set necessary to replicate your study findings to a stable, public repository and provide us with the relevant URLs, DOIs, or accession numbers. Please see http://www.bmj.com/content/340/bmj.c181.long for guidelines on how to de-identify and prepare clinical data for publication. For a list of recommended repositories, please see https://journals.plos.org/plosone/s/recommended-repositories. You also have the option of uploading the data as Supporting Information files, but we would recommend depositing data directly to a data repository if possible.

Please update your Data Availability statement in the submission form accordingly.

**The authors do not have the right to share any data information since the data is owned by a third-party organization, the Lebanese American University. Data can be shared upon a reasonable request from Ms. Perla Zgheib (perla.z.zgheib@gmail.com), Lecturer, Department of Social and Education Sciences, School of Arts and Sciences, Lebanese American University, Beirut, Lebanon**

**Human Ethics approval and Consent to participate declaration. In accordance with the**

**Declaration of Helsinki, all participants and their parents approved and signed the**

**informed consent to participate. Adolescent participants and their parents signed a**

**consent form. The document was sent home to their parents to sign it while**

**adolescents signed their consent form in class.**

**The consent was given by the Institutional Review Board (LAU IRB; reference number:**

**LAU.SAS.MJ1.28/Oct/2021).**

6. For studies involving third-party data, we encourage authors to share any data specific to their analyses that they can legally distribute. PLOS recognizes, however, that authors may be using third-party data they do not have the rights to share. When third-party data cannot be publicly shared, authors must provide all information necessary for interested researchers to apply to gain access to the data. (https://journals.plos.org/plosone/s/data-availability#loc-acceptable-data-access-restrictions)

For any third-party data that the authors cannot legally distribute, they should include the following information in their Data Availability Statement upon submission:
1) A description of the data set and the third-party source
2) If applicable, verification of permission to use the data set
3) Confirmation of whether the authors received any special privileges in accessing the data that other researchers would not have
4) All necessary contact information others would need to apply to gain access to the data

7. Please amend your authorship list in your manuscript file to include author “Sleiman El Hajj”.

**We apologize for the mishap; Dr Sleiman El Hajj is not an author on the manuscript.**

8. Your ethics statement should only appear in the Methods section of your manuscript. If your ethics statement is written in any section besides the Methods, please delete it from any other section.

**Done**.

9. Please include captions for your Supporting Information files at the end of your manuscript, and update any in-text citations to match accordingly. Please see our Supporting Information guidelines for more information: http://journals.plos.org/plosone/s/supporting-information.

**DONE. Thank you for the meticulous followup**

Reviewer #1:

Thank you for the opportunity to review this manuscript, which investigates the psychometric properties (i.e., factor structure, internal consistency, test-retest reliability, and convergent validity) of an Arabic version of the WHO-5 Well-Being Index among Lebanese adolescents.
I have three major concerns:
1. The study's contribution is somewhat unclear.

**The contribution of the study is to validate the use of a widely documented WHO-5 in an adolescent Arab speaking population. It is stated in the introduction “However, despite its widespread use, there is a surprising gap in validation studies within the Arab region, which is known to host one of the most youthful age structures globally”.**

2. Previous psychometric findings of the WHO-5 in adolescent samples are not sufficiently discussed.

**Noted. As per the suggestion, after listing some of the countries with validates WHO-5 in adolescents, we have discussed the psychometric findings “The scale has systematically shown a one factor structure similarly to the original version in adults with adequate convergent and divergent validity.”**

3. Some of the analyses could be expanded with more in-depth investigations.

**We changed the analyses and expanded the interpretation of the results. Please let us know if there is something else missing. Thank you.**

Please see my detailed comments below.
Title: The scale is typically referred to as the "WHO-5 Well-Being Index" rather than "WHO-5 wellbeing scale". I recommend adjusting the title (and throughout the manuscript) accordingly to ensure accessibility for interested readers.

**Thank you for the highlight, the naming has been amended in the title and the manuscript.**

Abstract: In the abstract, write out "WHO-5 Well-Being Index" in full when first mentioned, followed by the acronym in parentheses (i.e., WHO-5).

**Done.**

Introduction
There is insufficient discussion of previous research on the psychometric properties of the WHO-5. Please provide more information on studies that employed adolescent samples. See for instance, Adjorlolo & Anum (2021), Allgaier et al. (2012), Blom et al. (2012), Cosma et al. (2022), and Sischka et al. (2025). Discussing these findings will help contextualize your study.

**Thank you for the references. Some of these studies were added to better contextualize the current findings.**

Methods
Closely adhere to survey reporting guidelines (e.g., Turk et al., 2018, supplemental material S2). The following methodological details should be specified:
- Data Collection Method: Was the survey conducted online or via postal mail?

**The survey was conducted in person, in class for school students. This has been added accordingly.**

- Survey Accessibility: If online, was it open to everyone or restricted (e.g., password-protected)?

- Question Requirements: If online, did the survey include mandatory questions (e.g., Sischka et al., 2022)?
- Item Order: If online, were the items randomized or presented in a fixed order?

**To answer all three comments, we have clearly re-stated that the surveys were collected in class in person (and not online) and that the item order was fixed.**

- Recruitment Process: How was the survey advertised?

**The schools were randomly assigned by the Ministry of Education and the students within the appropriate age range were subsequently approached. This has been clarified in the manuscript**

- Incentives: Were any offered?

**No. No incentives were offered.**

- How was the sample size determined? Please provide details regarding your sample size considerations (see Giner-Sorolla et al., 2024).

**We have already included this information in the analytic strategy paragraph:**

**“We aimed to enroll a minimum of 100 adolescents following the recommendations of Mundfrom et al. of 3 to 20 times the number of the scale’s variables”
We have now included it in a separate paragraph for better clarity.**

**Giner-Sorolla et al. emphasize the importance of aligning sample sizes with the effect sizes researchers aim to detect. For CFA, this approach involves conducting a tailored power analysis that accounts for the number of factors and anticipated factor loadings. Given the brevity of the WHO-5 scale (five items loading on one factor), a sample size of 100 participants if often deemed sufficient to have enough statistical power (which is the same number we have already mentioned). We added the new reference in our paper and updated the text as follows:**

**We aimed to enroll a minimum of 100 adolescents following the recommendations of Mundfrom et al. of 3 to 20 times the number of the scale’s variables (30) and those of Giner-Sorolla et al (31).**

- You wrote: "Students were asked about their age and gender, then filled out the validated, publicly available Arabic version of the standardized scales for well-being (WHO-5), depression (PHQ-9), and anxiety (GAD-7), in that order." If this version of the WHO-5 has already been validated (which aspects?), please provide references supporting this claim. Additionally, clarify what the contribution of the current study is.

**The WHO-5 scale has already been validated in Arabic but in adult populations. The reference has already been used and was again provided and highlighted in the detailing of the WHO-5 scale.**
- You wrote “Some participants were tested twice for the same scales […]”. How were these participants selected? Was the second assessment available to all participants, or did only some opt to complete it?

**Only some opted to complete it, without any specific way to select those participants.**

- In the Analytical Strategy section, you state: "There were no missing responses in the dataset." However, in the Participants section, you wrote: "The sample consisted of 701 high school students […]. Among those participants, 681 students had complete data and were included in the final sample […]." This suggests that missing data were handled through listwise deletion. If this is the case, please state it explicitly. As missing data can be effectively handled using full information maximum likelihood (FIML) in AMOS – which is widely regarded as a best-practice method (e.g., Newman, 2014) – I strongly recommend employing this approach instead.
**We removed the sentence “There were no missing responses in the dataset”.**

**We added the results as follows:**

**Seven hundred adolescents participated in this study. The mean and standard deviation of the scores were as follows: WHO-5 wellbeing index (pre: 41.75 ± 22.61, post: 39.68 ± 21.24), PHQ-9 depression (11.10 ± 6.76), and GAD-7 anxiety (9.62 ± 5.50). The description of the categorization of participants according to their PHQ-9 and GAD-7 scores is summarized in “S1 Table”.**

**Table 1. Sociodemographic information and categorization of participants according to their depression and anxiety scores (N = 700)**

| **Age (years)** | **16.27 ± 1.11** |
| --- | --- |
| **Gender** |  |
| **Males** | **295 (42.1%)** |
| **Females** | **378 (54.0%)** |
| **Depression severity** |  |
| **No depression (PHQ-9 scores 0-4)** | **135 (19.3%)** |
| **Mild (PHQ-9 scores 5-9)** | **193 (27.6%)** |
| **Moderate (PHQ-9 scores 10-14)** | **146 (20.9%)** |
| **Moderately severe (PHQ-9 scores 15-19)** | **130 (18.6%)** |
| **Severe (PHQ-9 scores 20-27)** | **95 (13.6%)** |
| **Anxiety severity** |  |
| **No anxiety (GAD-7 scores 0-4)** | **147 (21.0%)** |
| **Mild (GAD-7 scores 5-9)** | **226 (32.3%)** |
| **Moderate (GAD-7 scores 10-14)** | **174 (24.9%)** |
| **Severe (GAD-7 scores ≥15)** | **153 (21.9%)** |

***Numbers might not round to the total N because of missing values.**

**We analyzed the database using the software R now.**

- The English WHO-5 response category reads as "At no time," but you wrote "Not at all." Please confirm whether this is an intended modification or a translation inconsistency.

**This is an inattention error, the scale was used with the “At no time” category indeed.**

- Please include the Arabic translation of the WHO-5 (i.e., items, response categories, and instructions) in the manuscript.

**This has been included in the supplement material S4 alongside with all available Arabic scales**

**The English version is included in S5**

- Besides gender invariance, consider testing for age-group invariance as well.

**We analyzed age invariance as requested by the reviewer. Results are added in table 2.**

- Please specify how the latent variable was scaled in the CFA model (e.g., Little et al., 2006).

**We added the following sentence to the analytic strategy paragraph:**

**“The latent variable was scaled in the CFA model by fixing the loading of the first item to 1.”**

- Add an explanation of the ICC in the Analytical Strategy section. Clearly specify which ICC coefficient was used, as different types exist with distinct assumptions and purposes (e.g., Aldridge et al., 2017).

**We added the following lines to the analytic strategy paragraph:**

**In order to assess the test-retest reliability of the WHO-5 scale, we calculated the intraclass correlation coefficient (ICC) using the reliability analysis option in SPSS v.27 to ensure the scale’s stability over time. We used the two-way mixed-effect model ICC. The ICC values vary between 0 and 1, with values >0.75 considered excellent.**

**Reference: Aldridge, V. K., Dovey, T. M., & Wade, A. (2017). Assessing test-retest reliability of psychological measures: Persistent methodological problems. European Psychologist, 22(4), 207–218.** [**https://doi.org/10.1027/1016-9040/a000298**](https://psycnet.apa.org/doi/10.1027/1016-9040/a000298)

Results
- Consider providing local model fit information in addition to global fit statistics (e.g., Kline, 2024). This would also allow for comparison with previous WHO-5 psychometric studies, many of which have reported high correlated residuals for items 1 and 2.

**We added the following to the results section:**

**To evaluate local fit, we examined the modification indices and residuals; the correlation between items 1 and 2 was borderline (= 10.05); therefore, we did not add a correlation between those two items.**

- Would it be possible to calculate effect size measures for measurement invariance (e.g., Nye et al., 2019; Gun et al., 2020)?

**We added the value of the effect size in the results section:**

**Cohen’s d = 0.307.**

- Please calculate item-level test-retest reliability coefficients (e.g., DeSimone, 2015) to provide a more nuanced view of item stability over time.
**We added the requested results as follows:**

**For the item-level test-retest reliability coefficients, the ICC values were as follows: item 1 (0.62 [95% CI .52; .69]), item 2 (0.61 [95% CI .51; .69]), item 3 (.65 [95% CI .57; .72]), item 4 (.63 [95% CI .54; .70]) and item 5 (.61 [95% CI .51; .69]).**

Discussion
The Results section states McDonald’s Omega = .83, but the Discussion section states McDonald’s Omega = .93. Please verify and correct this inconsistency.

**We apologize for the typo. We corrected it to 0.83.**

Minor points
- You wrote “Pearson test was used to correlate the WHO-5 scores with the other scales […]”. Please revise this to: "Pearson correlations were calculated to examine the association between the WHO-5 scores and the other scales."

**The Spearman test was actually used as statistical analyses were completely revamp based on reviewers comment. And the phrase was amended as per requested.**

- Please change the heading “Interclass Correlation” to “Intraclass Correlation”.
**Done as per suggested**

- This study addresses an important topic, and the manuscript is generally well-structured.

**Thank you, much appreciated**

- However, clarifying the study's contribution, ensuring consistency in reporting, and expanding key analyses (e.g., local model fit, test-retest at the item level, measurement invariance effect sizes) would significantly strengthen the paper. Additionally, adherence to survey reporting guidelines is crucial for transparency and reproducibility.

**We are keen on uplifting the manuscript and have added the missing results as suggested to better reflect consistency and expanded analyses.**

- I appreciate the authors' efforts and look forward to seeing the revised version.

**We thank for those remarks and hope the revised version is up to your expectations.**

*References*
- Adjorlolo, S., & Anum, A. (2021). Positive and negative psychosis risk symptoms among adolescents in Ghana. International Journal of Adolescence and Youth, 26(1), 307–320. <https://doi.org/10.1080/02673843.2021.1933110>

-Aldridge, V. K., Dovey, T. M., & Wade, A. (2017). Assessing test-retest reliability of psychological measures. European Psychologist, 22(4), 207-218. <https://doi.org/10.1027/1016-9040/a000298>
- Allgaier, A.-K., Pietsch, K., Frühe, B., Prast, E., Sigl-Glöckner, J., & Schulte-Körne, G. (2012). Depression in pediatric care: Is the WHO-Five Well-Being Index a valid screening instrument for children and adolescents? General Hospital Psychiatry, 34(3), 234–241. https://doi.org/10.1016/j.genhosppsych.2012.01.007
- Blom, E. H., Bech, P., Högberg, G., Larsson, J. O., & Serlachius, E. (2012). Screening for depressed mood in an adolescent psychiatric context by brief self-assessment scales - testing psychometric validity of WHO–5 and BDI-6 indices by latent trait analyses. Health and Quality of Life Outcomes, 10(1), 149. https://doi.org/10.1186/1477-7525-10-149
- Cosma, A., Költö, A., Chzhen, Y., Kleszczewska, D., Kalman, M., & Martin, G. (2022). Measurement invariance of the WHO–5 Well-Being Index: Evidence from 15 European countries. International Journal of Environmental Research and Public Health, 19(16), 9798. https://doi.org/10.3390/ijerph19169798
- DeSimone, J. A. (2015). New techniques for evaluating temporal consistency. Organizational Research Methods, 18(1), 133-152. https://doi.org/10.1177/1094428114553061
- Giner-Sorolla, R., Montoya, A. K., Reifman, A., Carpenter, T., Lewis Jr, N. A., Aberson, C. L., ... & Soderberg, C. (2024). Power to detect what? Considerations for planning and evaluating sample size. Personality and Social Psychology Review, 28(3), 276-301.
- Gunn, H. J., Grimm, K. J., & Edwards, M. C. (2020). Evaluation of six effect size measures of measurement non-invariance for continuous outcomes. Structural Equation Modeling, 27(4), 503-514. https://doi.org/10.1080/10705511.2019.1689507
- Kline, R. B. (2024). How to evaluate local fit (residuals) in large structural equation models. International Journal of Psychology, 59(6), 1293-1306. https://doi.org/10.1002/ijop.13252
- Little, T. D., Slegers, D. W., & Card, N. A. (2006). A non-arbitrary method of identifying and scaling latent variables in SEM and MACS models. Structural Equation Modeling, 13(1), 59-72. https://doi.org/10.1207/s15328007sem1301_3
- Newman, D. A. (2014). Missing data: Five practical guidelines. Organizational Research Methods, 17(4), 372-411. https://doi.org/10.1177/1094428114548590
- Nye, C. D., Bradburn, J., Olenick, J., Bialko, C., & Drasgow, F. (2019). How big are my effects? Examining the magnitude of effect sizes in studies of measurement equivalence. Organizational Research Methods, 22(3), 678-709.
- Sischka, P. E., Martin, G., Residori, C., Hammami, N., Page, N., Schnohr, C., & Cosma, A. (2025). Cross-National Validation of the WHO-5 Well-Being Index Within Adolescent -Populations: Findings From 43 Countries. Assessment. Advance online publication. https://doi.org/10.1177/10731911241309452
- Sischka, P. E., Décieux, J. P., Mergener, A., Neufang, K. M., & Schmidt, A. F. (2022). The impact of forced answering and reactance on answering behavior in online surveys. Social Science Computer Review, 40(2), 405-425. https://doi.org/10.1177/0894439320907067
- Turk, T., Elhady, M. T., Rashed, S., Abdelkhalek, M., Nasef, S. A., Khallaf, A. M., ... & Huy, N. T. (2018). Quality of reporting web-based and non-web-based survey studies: What authors, reviewers and consumers should consider. PloS one, 13(6), e0194239. https://doi.org/10.1371/journal.pone.0194239

Reviewer #2:

Thanks for sending the paper. I see that this validations study is more focus on statistical basis (CFA). However, there are other forms of validity and reliability. Please see the paper.
Arafat, S. Y., Chowdhury, H. R., Qusar, M. M. A. S., & Hafez, M. A. (2016). Cross cultural adaptation and psychometric validation of research instruments: a methodological review. Journal of Behavioral Health, 5(3), 129-136.
**We thank you for suggesting this paper. Based on the paper by Arafat et al. (2016), we considered other important types of validity and reliability besides the CFA. We did the measurement invariance, discriminant validity, concurrent validity, reliability using Cronbach’s alpha and McDonald’s omega and test-retest reliability. We think that all these results solidify our findings even more.**

Regarding the content and face validity:

**However, we did not include another scale to assess wellbeing in our survey. We added this idea as a limitation.**

**Thank you again.**

Reviewer #3:

- I strongly suggest that the authors consider the addition of a descriptive table to show the sample profile, using variables of individual characteristics, such as gender, age, sexual orientation and grade level.
**A descriptive table (Table 1 now) was added as suggested. We added the age and gender variables only since we did not ask about sexual orientation.**

- Regarding the analytic strategy, I strongly recommend the use of R or JASP to run the analysis.

**As per reviewer’s suggestion, we ran the analyses using the R software.**

- Furthermore, considering that the WHO-5 is a 6 point likert scale, I suggest to evaluate the multivariate normality assumption of SEM with Mardia’s test, and in case of violations in multivariate normality, I suggest to use the Weighted Least Squares Mean and Variance adjusted estimator (WLSMV), due to the ordinal nature of the items, instead of non-parametric bootstraps.

**We added the information regarding the Mardia’s test and we did the analysis using the R software and the WLSMV estimator.**

 -In the “Further analyses” topic, I suggest to use the Shapiro-Wilks or Kolmogorov-Smirnov test to assess the assumption of normality distribution, in order to use the t-test and the Pearson’s correlation index.
- It seems that the authors know the relevance of assumptions of the statistical analysis, but the statistical procedure lacks on the correction of the violations of these assumptions.
**Thank you for raising the issue, to better answer to the two previous comments, we used the Shapiro-Wilk test, which showed a significant p value, showing non normal distribution.**

**Therefore, we used the Mann-Whitney and Spearman tests for the remainder of the analysis as suggested.**

- The “Participants” topic is duplicated (it appears on methods and results), and it seems that this section in the “results” is referring to the test-retest reliability results.

**This has been amended.**

- It’s strongly recommended to use the Student’s t test, Generalized Linear Models (GLM) or Mann-Whitney tests to compare groups, in order to assess whether the results are different between measurement times.

**We used the Mann-Whitney test as suggested.**

-The test-retest reliability can be tested using linear correlation between the results (pre and post), with strong correlations indicating good results.

**We added the results as suggested:**

**The correlation between the total scores pre and post was r = 0.64; p < 0.001 (moderate to strong effect).**

- In “confirmatory factor analysis of the WHO-5 scale”, the article mentions the composite reliability using the alpha and omega letters. It seems to be a typing error, these are not composite reliability indexes, but internal consistency indexes, and composite reliability is another one, calculated using factor loadings, it needs to be corrected.

**We corrected the terminology to “internal consistency”.**

- Regarding the “gender invariance”, it’s not clear why to compare groups using t test by gender, since the factorial invariance is measured using CFA models, and not the general score of the instrument (table 2 is the correct for evaluating factorial invariance).
**We apologize for the confusion.**

**We did the measurement invariance to check if the scale measures the same construct in the same way across different groups. After establishing measurement invariance, we compared the total scores between the two genders to see if there is any mean difference between them. This is a legitimate step as it tests whether the overall wellbeing score differs between genders.**

- I suggest the authors to avoid very small topics, as interclass correlation and concurrent validity.

**Inasmuch as we agree regarding the small paragraphs, however, we would like to keep them separate so the reader can easily detect the information based on the subtitle.**

**If the rational does not align with the rigorous review process, we will be more than happy to merge the paragraphs together. Thank you for your understanding.**

- In the discussion, the authors mentions the “divergent validity”, but it's not clear how it was measured.

**We apologize for this mistake. The term was changed to “concurrent validity”.**
