# Supplementary material for: Psychometric validation of an Arabic version of the WHO-5 wellbeing index among Lebanese adolescents
Source: PLoS One. 2025 Jul 17;20(7):e0317644. doi: 10.1371/journal.pone.0317644 (PMC12270098; doi:10.1371/journal.pone.0317644)
Supplement: Attachment — Submitted filename: Psychomteric_WHO5_ReviewerResponse_050625.docx [file pone.0317644.s005.docx]

**Dearest Editor,**

**Thank you and the team for the rigorous followup**

**We are hereby providing our comments and point-by-point review in bold below. Changes are highlighted in the manuscript to facilitate tracking**

**We do hope the uplifted version suits the publication criteria of PlosOne.**

**Very much looking forward to the publication**

**Myriam EL khour-Malhame**

Dear Dr. El Khoury Malhame,

Thank you for submitting your manuscript to PLOS ONE. After careful consideration, we feel that it has merit but does not fully meet PLOS ONE’s publication criteria as it currently stands. Therefore, we invite you to submit a revised version of the manuscript that addresses the points raised during the review process.

==============================

The manuscript has been improved through changes made on the most recent revision. However, there are still methodological considerations (see Reviewer 1 comments) that require clarification prior to acceptance.

Prior to acceptance, the manuscript should include the following changes:

1. The estimation methods must be clearly specified.

**Parameter estimates were obtained using the robust maximum likelihood method.**

2. Explicitly state the ICC model used and whether your aim was to assess consistency or absolute agreement.

**We used the ICC(3,1), i.e., two-way mixed-effect model ICC, consistency type and single measures.**

**This was clarified in the statistical analysis paragraph.**

3. Clarify whether the internal consistency of each instrument cited in the manuscript were calculated using the current sample or drawn from previous studies.
==============================
We look forward to receiving your revised manuscript.

Kind regards,

Janna Metzler
Academic Editor
PLOS ONE

**Reviewer #1:**

Thank you for the opportunity to re-review this manuscript. Some aspects have been improved, but there are still several areas that require further consideration.
- In the Methods section, three different CFA estimation methods are mentioned (i.e., WLSMV, maximum likelihood, robust maximum likelihood; p. 7, lines 5, 6, and 14). Please clarify which estimator was actually used and revise the description accordingly to accurately reflect your analytic procedure. Misreporting estimation methods can cause confusion and undermine the reproducibility of your results.

**We apologize for the confusion. We kept this sentence:**

**Parameter estimates were obtained using the robust maximum likelihood method.**

- Regarding effect sizes, I was referring to measurement invariance effect sizes that indicate the magnitude of non-invariance (e.g., Gunn et al., 2020; Nye et al., 2019), not effect sizes for mean differences. That said, this was only a suggestion.

**We apologize for the misunderstanding. We added the results as advised according to the references suggested. Thank you.**

- The phrase "two-way mixed-effect model ICC" is not specific enough to determine exactly which ICC type was used. SPSS provides several ICC variants under this model, depending on whether one is assessing single vs. average measures, and consistency vs. absolute agreement (see also Shrout & Fleiss, 1979). To enhance transparency and allow proper interpretation, please explicitly state the ICC model used (e.g., ICC(3,1)) and whether your aim was to assess consistency or absolute agreement. This level of detail is important for evaluating the reliability of the WHO-5 across time points.

**SPSS provides several models: two-way mixed, two-way random, and one-way random.**

**For the type, it can be either consistency or absolute agreement.**

**We used the ICC(3,1), i.e., two-way mixed-effect model ICC, consistency type and single measures.**

**This was clarified in the statistical analysis paragraph.**

- Please also clarify whether the internal consistency estimates for the GAD-7 and PHQ-9 reported in the Measures section were calculated using the current sample, or if they were taken from previous studies. If the latter is the case, please additionally report the internal consistency values based on your current sample.

**We added the Cronbach’s alpha values in this study.**

The manuscript should be thoroughly proofread, as some sentences remain awkwardly phrased and/or vague/ambiguous. A few examples:
- “However, some missing data was found in the other variables (age, gender, etc.) and were not replaced.” Please rephrase into something like “Some missing data were present in demographic variables such as age and gender. These missing values were not imputed and were handled using listwise deletion in the relevant analyses.”

**Sentence rephrased as suggested by the reviewer.**

- “Normality was not verified since the p value of the Shapiro-Wilk test turned out significant (p < 0.001).” Please rephrase into something like “The distribution of the WHO-5 scores was not normal, as indicated by a significant result from the Shapiro-Wilk test (p < .001).”
**Sentence rephrased as suggested by the reviewer.**

References
Aldridge, V. K., Dovey, T. M., & Wade, A. (2017). Assessing test-retest reliability of psychological measures: Persistent methodological problems. European Psychologist, 22(4), 207–218. https://doi.org/10.1027/1016-9040/a000298
Gunn, H. J., Grimm, K. J., & Edwards, M. C. (2020). Evaluation of six effect size measures of measurement non-invariance for continuous outcomes. Structural Equation Modeling, 27(4), 503-514. https://doi.org/10.1080/10705511.2019.1689507
Nye, C. D., Bradburn, J., Olenick, J., Bialko, C., & Drasgow, F. (2019). How big are my effects? Examining the magnitude of effect sizes in studies of measurement equivalence. Organizational Research Methods, 22(3), 678-709. https://doi.org/10.1177/1094428118761122
Shrout, P. E., & Fleiss, J. L. (1979). Intraclass correlations: Uses in assessing rater reliability. Psychological Bulletin, 86(2), 420–428. <https://doi.org/10.1037/0033-2909.86.2.420>

**Thank you for sending the references. We have incorporated them in our paper.**

**We hope the revised version is up to your expectations. Thank you again for your time and efforts**.
